# The impact of smoking and diabetes on periodontitis grade: A retrospective cross-sectional observational study

**DOI:** 10.1186/s12903-026-10095-x

**Published:** 2026-10-31

**Authors:** Gina Steinbrenner, Neelam Lingwal, Nihad El Sayed, Peter Eickholz

**Affiliations:** 1 Goethe-University Frankfurt am Main, Center for Dentistry and OralMedicine (Carolinum), Department of Periodontology, Theodor-Stern-Kai 7 (Haus 29), Frankfurt am Main, 60596 Germany; 2 Goethe-University Frankfurt am Main, Center for Dentistry and Oral Medicine (Carolinum), Theodor-Stern-Kai 7 (Haus 29), Frankfurt am Main, 60596 Germany

**Keywords:** Periodontitis grade A, B, C, Smoking, Diabetes, Classification

## Abstract

**Background:**

Evaluation of the amount of periodontitis patients whose grade is determined by smoking and/or diabetes.

**Methods:**

All charts of patients scheduled for active periodontal treatment at Goethe University Frankfurt, Germany, Dept. of Periodontology from November 2004 on were screened until data saturation was reached, and 402 charts met the inclusion criteria. Charts were analysed and patients reclassified according to the 2018 classification of periodontal diseases. Grade A/B was also assigned according to Dietrich et al. 2019.

**Results:**

Four-hundred-two patients (age: 56.9 ± 11.4 years; 208 females; 68 active smokers; 22: 1–9 cigarettes/day; 46: ≥10 cigarettes/day; 29 diabetics, 18: HbA1c < 7%, 11: HbA1c ≥ 7%; 7 stage II, 234 stage III, 161 stage IV, 0 grade A, 179 grade B [Tonetti et al. 2018], 10 grade A, 169 grade B [Dietrich et al. 2019], 223 grade C) contributed 9,318 teeth and 221 dental implants. No heavy smoker (≥ 10 cigarettes/day) suffered from HbA1c ≥ 7%. Generalised stage III periodontitis grade C was exclusively determined by smoking in 7 and by diabetes in 3 cases.

**Conclusions:**

In this convenience periodontitis sample in generalised stage III periodontitis, grade C was rarely determined exclusively by smoking (7 cases [7%]) or diabetes (3 cases [3%]). Only 10 patients were assigned to different grades according to Dietrich et al. 2019 (2.5%).

**Trial registration:**

March 11, 2024 registered in the German Register of Clinical Studies (Deutsches Register Klinischer Studien: DRKS) DRKS00033633.

## Introduction

Use of systemic amoxicillin and metronidazole adjunctive to subgingival instrumentation (SI) results in significantly better clinical results than exclusive SI [1–6]. However, uncritical use of systemic antibiotics carries profound risk, in particular antimicrobial resistance. Increase of antimicrobial resistances is closely related to frequency of use of antibiotics [7]. Consequently, systemic antibiotics shall be used restrictively in particular in the treatment of non-life-threatening diseases (e.g., periodontitis) [8, 9]. The diagnosis periodontitis, generalized stage III, grade C shows the greatest clinical benefit from adjunctive amoxicillin and metronidazole [10]. However, progression rate of grade C could be determined by uncontrolled diabetes mellitus and/or heavy smoking [11].

The British Society of Periodontology (BSP) has criticized the bone loss/age threshold of < 0.25 to assign grade A and proposed higher margins (Dietrich et al., 2019). Physiological bone levels range from 1.0 to 3.0 mm apical to the CEJ (Chapple et al., 2018). This means that periodontitis starts at a distance > 3.0 mm to the CEJ. For assignment of grade A low relative bone loss (BL)/age coefficients are required (< 0.25). Thus, we need low relative BL in individuals with high age. Does the BSP threshold make a substantial difference in the assignment of grade A or B?

Thus, this retrospective cross-sectional observational study was designed to assess:

- the frequencies of periodontitis diagnoses in the sample of patients undergoing comprehensive periodontal treatment at the department of periodontology of Goethe University Frankfurt/Main, Germany.
- the percentage of patients that meet the subgroup criteria identified in the exploratory analysis of the ABPARO trial to benefit most from systemic amoxicillin and metronidazole adjunctive to SI (periodontitis, generalized stage III, grade C) [10].
- the percentage of patients whose grade C is determined by diabetes and/or smoking and not primarily by bone loss age coefficient.
- the effect of bone loss age coefficient 0.25 [12] or 0.5 [13] on the frequency of grade A.

## Materials and methods

### Patients

All charts of patients for whom comprehensive periodontal treatment was applied for at the statutory health insurance since April 2005 and of patients of Prof. Eickholz’s intramural private practice who received comprehensive periodontal treatment since October 2004 at the Department of Periodontology of Goethe-University Frankfurt/Main, Germany were screened in portions of 30 to 50 charts. All patients have given written consent that their anonymised data may be evaluated for scientific purposes.

### Inclusion criteria

- Application for comprehensive periodontal treatment by the Department of Periodontology of Goethe-University at statutory insurance since April 2005 or comprehensive periodontal treatment at Prof. Eickholz’s private practice since October 2004.
- Complete periodontal charting prior to treatment (baseline examination; T0) with probing pocket depths (PPD) and clinical attachment loss (CAL) at 6 sites/tooth (PCPUNC 15, Hu-Friedy, Chicago, USA), bleeding on probing (BOP), furcation involvement (FI) (Hamp et al., 1975) at all furcation entrances of multi-rooted teeth. FI was exclusively assessed clinically using a Nabers probe (PQ2N, Hu-Friedy, Chicago, USA). In patients exhibiting profuse BOP, the percentage was set 100%.
- Self report on nicotine consumption (number of cigarettes/day) at T0.
- Self report on suffering from diabetes and HbA1c (%) at T0.
- Radiographs of all teeth not older than 12 months at T0 (panoramic or complete sets of periapical radiographs).

### Exclusion criteria

- missing data.

The trial was approved by the Institutional Review Board for Human Studies of the Medical Faculty of Goethe-University Frankfurt/Main (2023 − 1591) and was in March 11, 2024 registered in the German Register of Clinical Studies (Deutsches Register Klinischer Studien: DRKS) DRKS00033633.

### Analysis of patients‘ charts

For each patient the following data were extracted from the patients‘ charts:

- Age at T0.
- Self-reported smoking (non-smoker/former smoker; moderate smoker: <10 cigarettes/day; heavy smoker: ≥10 cigarettes/day) (T0).
- Self-reported diabetes mellitus, HbA1c (%) (T0).
- Gingival Bleeding Index (GBI [14]) and Plaque Control Record (PCR [15]) at T0 or the first appointment of step 1 up to 6 months after T0.
- Number of teeth and dental implants (T0).
- Amount of shallow (PPD ≤ 3 mm), moderate (PPD 4–5 mm) and deep (PPD ≥ 6 mm) pockets (T0).

### Analysis of radiographs

Ten sets of radiographs (five panoramic and five sets of periapical radiographs) from 10 patients unrelated to this trial were used to calibrate the investigator (G.S.), who assessed grade by relative bone loss/age coefficient. P.E. and G.S. independently assessed relative bone loss/age coefficient and accordingly assigned grades using the 10 sets of radiographs [12]. Agreement was assessed by Cohen’s kappa [16]. The same approach had been used in earlier analyses [10].

To estimate tooth loss due to periodontal reasons and to assess the bone loss/age quotient G.S. analyzed radiographs (panoramic or complete sets of periapical radiographs) that had been obtained up to 12 months prior to T0. All radiographs were digitalized using a computer program (SIDEXIS nextGeneration 1.51, Sirona, Bensheim, Germany) and a flatbed scanner (Epson Perfection V850 Pro, Epson, Suwa, Nagano, Japan) with 600 dpi resolution and 8-bit grey values. The image files were stored as TIFF files and analysed using the computer program SIDEXIS and a 22’ flat screen (LCD Display P24-9 TE, Fujitsu, Tokio, Japan) in a particular room under exclusion of natural or artificial light except the screen.

Regarding tooth loss due to periodontal reason third molars were not taken into account. If the alveolar crest in tooth gaps was located > 33% of the root length of adjacent teeth the reason for loss of the respective teeth was classified as periodontal.

Based on baseline examination and radiographs each patient was retrospectively assigned a diagnosis (e.g., periodontitis, generalized stage III, grade B) according to the 2018 classification [11]. Stage and extension were assigned due to interdental CAL, tooth loss according to periodontal reasons and complexity factors (stage III: PPD, FI, deep intrabony defects; stage IV: less than 20 teeth, less than 10 pairs of occluding teeth). Grade was assigned according bone loss/age coefficient, self-reported smoking and diabetes [12]. Further, grade A/B were also assigned according to Dietrich et al. 2019 [13].

### Statistical analysis

Assignment of grade A [12] is very rare in periodontitis patients treated at the Department of Periodontology of Goethe University Frankfurt/Main [17, 18] and also in the general population where grade A was observed only in stage I periodontitis of young adults (35–44 years) and mostly in stage I periodontitis of young seniors (65–74 years) [19]. Not knowing how many patients will be assigned to grade A according to Dietrich et al. 2019 [13], pragmatically rather than based on a formal a priori power calculation the decision was made to include a great number of patients, i.e., 400 as the authors state that the expected number of grade A patients was unknown beforehand.

The patient was defined as the statistical unit and the main outcome variable is frequency of grade A, B, C assigned according to heavy smoking, HbA1c ≥ 7% and different determinants [12, 13]. Statistical analyses were performed using PC programs (Systat™ for Windows Version 13, Systat Inc. Evanston, IL, USA). Cohen’s and weighted kappa were calculated using MedCalc Software Ltd. Inter-rater agreement (Kappa). https://www.medcalc.org/en/calc/kappa.php (Version 23.3.7; accessed October 12, 2025). Patient characteristics were described as absolute and relative frequencies (binomial and categorial variables) or means±standard deviations, medians and lower/upper quartils (continuous variables). BOP, GBI, PCR, percentages of PPD (≤3, 4–5, ≥ 6 mm), numbers of teeth and dental implants were tested for normal distribution using Kolmogorow-Smirnov test. All variables not determining grade (age, smoking, diabetes) were compared between grades A, B, C [13] using Kruskal-Wallis test. We planned to calculate approximately 15 comparisons. Due to this multiple testing the p value was set to < 0.001. After Bonferroni adjustment for 15 comparisons the significance level would be 0.0033 and, thus, robust below 0.05 [20].

The recommendations of the Strengthening the Reporting of Observational Studies in Epidemiology (STROBE) guidelines were applied for reporting [21].

## Results

### Patients

After the last portion of patients’ charts had been screened for eligibility, we retained 402 instead of 400 charts and decided to include all 402 qualifying charts. Thus, 402 patients (56.9 ± 11.4 years) were included into this analysis of which 208 (53%) were female. Only 11 (3%) patients suffered from uncontrolled diabetes (HbA1c ≥ 7%) and 46 (11%) were heavy smokers (≥ 10 cigarettes/day) and, thus, qualifying for grade C (Table 1). All patients contributed 9,318 teeth and 221 dental implants to this analysis. Table 2 provides periodontal parameters. Percentage of sites with PPD ≤ 3 mm and 4-5 mm was significantly lower for grade C than B. Percentage of sites with PPD ≥ 6 mm was significantly higher for grade C than grade A and B [13], whereas, there was no significant difference between grade A and B. Table 3 provides the frequency of diagnoses according to stage, extent and grade. None of the heavy smokers suffered from uncontrolled diabetes. Just in 23 cases, generalized stage III periodontitis coincided with heavy smoking (20) and/or uncontrolled diabetes (3) (Table 4). Of these cases, 10 with a bone loss/age coefficient ≤ 1 (grade B) heavy smoking (7) or uncontrolled diabetes (3) determined grade C. In all other generalized stage III, grade C cases (86) heavy smoking or uncontrolled diabetes coincided with bone loss/age coefficient > 1 (grade C).

**Table 1:** Patient characteristics at T0 (SD: standard deviation; median: M; lower quartile: LQ; upper quartile: UQ). No Grade A according to Tonetti et al. 2018 [12]

	All	Grade A (Dietrich et al. 2019) [13]	Grade B (Dietrich et al. 2019) [13]	Grade B (Tonetti et al. 2018) [12]	Grade C
Total number	402	10	169	179	223
Females [n (%)]	208 (52)	6 (60)	91 (54)	97 (54)	111 (50)
Age [years]	56.9 ± 11.4	67.4 ± 10.6	60.9 ± 10.1	61.2 ± 10.2	53.5 ± 11.2
(Mean ± SD; M, LQ/UQ)	56.5, 49/65	65.5, 60/78	61, 55.8/68	61, 56/68	53, 46/60.8
Diabetes					
No diabetes [n (%)]	373 (93)	10 (100)	161 (95)	171 (96)	202 (91)
HbA1c < 7% [n (%)]	18 (4)	0	8 (5)	8 (4)	10 (4)
HbA1c ≥ 7% [n (%)]	11 (3)	0	0	0	11 (5)
Smoking					
Non-smokers [n (%)]	334 (83)	10 (100)	163 (96)	173 (97)	161 (72)
< 10 cigarettes/day [n (%)]	22 (6)	0	6 (4)	6 (3)	16 (7)
≥ 10 cigarettes/day [n (%)]	46 (11)	0	0	0	46 (21)

**Table 2:** Periodontal characteristics at T0 (SD: standard deviation; median: M; lower quartile: LQ; upper quartile: UQ; probing pocket depth: PPD). No Grade A according to Tonetti et al. 2018 [12]

	All	Grade A (Dietrich et al. 2019) [13]	Grade B (Dietrich et al. 2019) [13]	Grade B (Tonetti et al. 2018) [12]	Grade C
Total number	402	10	169	179	223
Bleeding on probing [%] (Mean ± SD; M, LQ/UQ)	37.4 ± 28 28, 18/47	35.5 ± 35 22^A^, 20/31	32.9 ± 24.8 25^A^, 18/38.3	33.1 ± 25.4 25, 18/37.8	40.9 ± 29.6 31, 18.3/56
Gingival bleeding index [%] (Mean ± SD; M, LQ/UQ)	9.2 ± 11.4 6, 2/12	13.1 ± 12 11, 5/18	8.5 ± 9.5 6, 2/12	8.8 ± 9.6 6, 2/12	9.7 ± 12.7 6, 2/13
Plaque control record [%] (Mean ± SD; M, LQ/UQ)	45.5 ± 19.6 45, 33/58	45.9 ± 20.8 43.5, 29/59	43.8 ± 17.2 44, 34/52	43.9 ± 17.3 44, 33.3/52	46.5 ± 21.4 45, 32.3/61
Sites with PPD 1–3 mm [%] (Mean ± SD; M, LQ/UQ)	70.5 ± 18 74.5, 60.7/84	80.2 ± 4.4 80.4 , 77.2/83.3	78 ± 13.2 80.7 ^A^, 71.4/87.4	78.1 ± 12.9 80.7 ^A^, 71.8/87.1	64.4 ± 19.2 68, 51.2/80.2
Sites with PPD 4–5 mm [%] (Mean ± SD; M, LQ/UQ)	23.2 ± 12.1 20.7, 14/30	19 ± 4.3 18.9 , 14.7/22	19.9 ± 10.7 18.6 ^A^ , 11.9/25.7	19.9 ± 10.4 18.6 ^A^, 12.2/25.4	25.9 ± 12.7 23.6, 16/34.3
Sites with PPD ≥ 6 mm [%] (Mean ± SD; M, LQ/UQ)	7.3 ± 9.9 3.3, 0.7/10.1	1.9 ± 2.3 1.5 ^A^, 0/2.6	3 ± 4.5 1.6 ^A^ , 0/3.2	3 ± 4.4 1.6 ^A^, 0/3.2	10.8 ± 11.7 7.7, 2.5/14.3
Number of teeth without wisdom teeth (total n/ Mean ± SD; M, LQ/UQ)	8,999 22.4 ± 5.2 24, 20/27	225 22.5 ± 5.1 23.5, 18/27	3,913 22.5 ± 4.6 24, 19/26	4,026 22.5 ± 4.6 24, 19/26	4,973 22.3 ± 5.6 24, 20/27
Number of teeth with wisdom teeth (total n/ Mean ± SD; M, LQ/UQ)	9,318 23.2 ± 5.6 24, 20/27	230 23 ± 5.1 25, 19/27	3,801 23.2 ± 4.9 24, 20/27	4,143 23.1 ± 4.9 24, 20/27	5,175 23.2 ± 6 25, 20/27
Number of dental implants (total n/Mean ± SD; M, LQ/UQ)	221 0.6 ± 1.4 0, 0/0	6 0.6 ± 1.9 0, 0/0	140 0.8 ± 1.7 0 ^A^, 0/1	146 0.8 ± 1.7 0 ^A^, 0/1	75 0.3 ± 1 0, 0/0

^A^ significantly different from grade C; *p* < 0.001

**Table 3:** Diagnoses at T0: periodontitis according to stage and grade [n (%)]; MIP: molar incisor pattern [13]

Stage	Extent	Grade A	Grade B	Grade C	total
II	localized	0	1 (0.2)	0	1 (0.2)
II	generalized	0	5 (1.2)	0	5 (1.2)
II	MIP	0	1 (0.2)	0	1 (0.2)
III	localized	4 (1)	50 (12.4)	37 (9.2)	91 (22.6)
III	generalized	1 (0.2)	42 (10.4)	96 (23.9)	139 (34.6)
III	MIP	0	1 (0.2)	3 (0.7)	4 (1)
IV	localized	4 (1)	24 (6)	6 (1.5)	34 (8.5)
IV	generalized	1 (0.2)	45 (11.2)	81 (20.1)	127 (31.5)
total		10 (2.5)	169 (42)	223 (55.5)	402

**Table 4:** Patients according to stage and extent for stage III as well as criteria determining grade C (BL: bone loss; MIP: molar incisor pattern)

Stage	Extent	total	BL/age index > 1	HbA1c ≥ 7%	≥ 10 cigarettes/day
II		7	0	0	0
III	localized	91	30	2	7
III	generalized	139	86	3	20
III	MIP	4	3	0	0
IV		161	78	6	19
total		402	197	11	46

Whereas none of 402 patients were assigned to grade A according to the 2018 Classification of Periodontal and Peri-implant Diseases and Conditions [12], 10 patients (2%) were assigned to grade A according to Dietrich et al. 2019 [13] (Table 1). Accounting for all grades, agreement between assignment according to Tonetti et al. 2018 and Dietrich et al. 2019 was excellent agreement [16] or very good [22] (weighted kappa: 0.952; standard error 0.014; 95% confidence interval: 0.924–0.980). Comparing just assignment of grades A and B, agreement was poor (Cohen’s kappa: 0).

### Rater agreement

P.E. judged the same tooth as having the most severe relative bone loss as G.S. in eight patients (80%). The respective grade assignment according to bone loss/age index was similar in nine patients (Cohen’s kappa 0.615; 95% confidence interval − 0.045-1) representing fair to good agreement [16] or good agreement [22].

## Discussion

The objective of this retrospective cross-sectional observational study was to evaluate the frequencies of periodontitis diagnoses in the sample of patients undergoing comprehensive periodontal treatment at the Department of Periodontology of Goethe University Frankfurt/Main, Germany. In particular the following should be assessed:

- the frequencies of periodontitis diagnoses in the sample of patients undergoing comprehensive periodontal treatment at the Department of Periodontology of Goethe University Frankfurt/Main, Germany,
- the percentage of patients that meet the subgroup criteria identified in the exploratory analysis of the ABPARO trial to benefit most from systemic amoxicillin and metronidazole adjunctive to SI (periodontitis, generalized stage III, grade C) [10].
- the percentage of patients whose grade is determined exclusively by diabetes and/or smoking,
- the effect of bone loss age coefficient 0.25 [12] or 0.5 [13] on the frequency of grade A/B.

All charts of patients scheduled for active periodontal treatment at Goethe University Frankfurt, Germany, Dept. of Periodontology were screened until 402 could be included contributing 9,318 teeth and 221 dental implants. Charts were analysed and patients reclassified according to the 2018 Classification of Periodontal Diseases [12]. Grade A/B was also assigned according to Dietrich et al. 2019 [13]. No heavy smoker (≥ 10 cigarettes/day) suffered from HbA1c ≥ 7%. In this convenience periodontitis sample in generalized stage III periodontitis, grade C was rarely determined exclusively by smoking (7/7%) or diabetes (3/3%). Only 10 patients were assigned to different grades according to Dietrich et al. 2019 (2.5%).

Assignment of grade A [12] is very rare in periodontitis patients treated at the Department of Periodontology of Goethe University Frankfurt/Main [17, 18] and also in the general German population where grade A was observed only in stage I periodontitis of young adults (35–44 years) and mostly in stage I periodontitis of young seniors (65–74 years) [19]. The British Society of Periodontology has criticized the upper threshold of grade A (BL/age coefficient < 0.25). They wrote that if grade A is defined as a BL/age coefficient of < 0.25, few patients would be classified as grade A [13]. For example, a 60-year-old patient with no more than 20% bone loss on all affected teeth would be classified as grade B (moderate rate of progression). In periodontally healthy individuals the assignment of periodontitis grade is no option. Physiological bone levels range from 1.0 to 3.0 mm apical to the CEJ (Chapple et al., 2018). This means that periodontitis starts at a distance > 3.0 mm to the CEJ. For assignment of grade A low BL/age coefficient is required (< 0.25). Thus, low relative bone loss is required in individuals with high age. Choosing a tooth with a long root (length of e.g., 16 mm; maxillary canine) relative radiographic BL would be 19.4% ([16 − 3]/16 mm). To result in a BL/age coefficient < 0.25 the respective patient would have to be 75 years or older. The oldest individual in this study actually is 78. However, when determining grading by indirect evidence the respective tooth with the most severe relative radiographic bone loss has to be chosen. This is unlikely to be the tooth with the longest but more likely the tooth with the shortest root (the shorter the root with given distance bone to CEJ, the higher is relative BL). Hence, the lack of grade A in this study is due to a lack of elderly individuals (> 75 years of age) [17]. However, due to more than 3 mm BL the respective 78 years old patient is not classified grade A but B [12]. According to Dietrich et al. 2019 she or he may be assigned grade A [13].

The EFP S3 level clinical guideline does not recommend the routine use of systemic antibiotics as adjunct to SI in patients with periodontitis due to concerns about patient’s health and the impact of systemic antibiotic use to public health. However, the adjunctive use of specific systemic antibiotics may be considered for specific patient categories (e.g., generalized periodontitis Stage III in young adults) [9]. The adolopement of this guideline by the German Society of Periodontology extends this open recommendation to stage IV periodontitis [8]. What are young adults in this context? A recent explanatory reanalysis of the ABPARO-Study identified patients suffering from periodontitis, generalized stage III, grade C as subgroup that benefits the most from systemic amoxicillin and metronidazole adjunctive to SI. For stage IV periodontitis the benefit did not reach the defined level of clinical relevance [10]. Thus, periodontitis, generalized stage III was confirmed and “young adults” has become more concrete with grade C. However, grade C may be assigned in patients with a bone loss/age coefficient ≤ 1 (grade B) not due to rapid progression but due to heavy smoking or uncontrolled diabetes. Thus, this retrospective analysis was performed to assess the proportion of patients who are assigned to grade C exclusively due to heavy smoking or uncontrolled diabetes. This was the case due to heavy smoking in 7 or uncontrolled diabetes in 3 cases. In all other generalized stage III, grade C cases (86) heavy smoking or uncontrolled diabetes coincided with bone loss/age coefficient > 1. Of all generalized stage III, grade C periodontitis cases (*n* = 96), 7% met the subgroup criteria identified in the exploratory analysis of the ABPARO trail [10] to benefit most by systemic amoxicillin and metronidazole exclusively due to heavy smoking and 3% due to uncontrolled diabetes.

Cohen’s kappa reveals an “excellent” or “very good” weighted kappa (0.952) for the agreement between the Tonetti et al. 2018 [12] and Dietrich et al. 2019 [13] grade definition (bone loss/age coefficient) overall. However, the kappa value for distinguishing between grades A and B is reported as 0. This is statistically plausible with the very rare category of grade A according to Dietrich et al. 2019 [13] (10 of a total of 179).

However, within this convenience sample of patients scheduled for comprehensive periodontal treatment using the BL/age coefficient of < 0.5 does not make a big difference [13]. Using 0.5 only 10 of 402 (2.5%) patients are assigned to grade A. This may be due to the fact that more severe periodontal cases are referred to the Department of Periodontology of Goethe-University Frankfurt/Main.

What are the limitations of this analysis? First of all, this is a retrospective convenience sample study with a high risk of bias. In particular at an academic department of periodontology an overrepresentation of severe diagnoses would be plausible. With more mild to moderate cases the amount of diagnoses for grade A according to Dietrich et al. 2019 [13] plausibly will increase. For the assessment of population representative frequencies of diagnoses and an appreciation of the different thresholds for grade A/B, analyses of population representative epidemiologic studies are required. HbA1c values were not verified by sampling. They are based on self report. However, the 2018 Classification of Periodontal and Peri-implant Diseases and Conditions does not require blood analyses for assessment of HbA1c but self-report [12].

Within the limitations of the present study, the following conclusions regarding frequency of periodontal diagnoses with respect to grade may be drawn from this convenience periodontitis sample:

- In generalized stage III periodontitis, grade C was rarely determined exclusively by smoking (7 cases [7%]) or diabetes (3 cases [3%]).
- Only 10 patients were assigned to different grades according to Dietrich et al. 2019 (2.5%).

## Data Availability

The data of this study are available from the corresponding author upon reasonable request.
